# Decreased transcriptional corepressor p107 is associated with exercise‐induced mitochondrial biogenesis in human skeletal muscle

**DOI:** 10.14814/phy2.13155

**Published:** 2017-03-07

**Authors:** Debasmita Bhattacharya, Mia Ydfors, Meghan C. Hughes, Jessica Norrbom, Christopher G. R. Perry, Anthony Scimè

**Affiliations:** ^1^ Stem Cell Research GroupMolecularCellular and Integrative PhysiologyFaculty of HealthYork UniversityTorontoCanada; ^2^ MolecularCellular and Integrative PhysiologyFaculty of HealthYork UniversityTorontoCanada; ^3^ Department of Physiology and PharmacologyKarolinska InstitutetStockholmSweden

**Keywords:** Exercise, human, mitochondrial biogenesis, OXPHOS, Rb family

## Abstract

Increased mitochondrial content is a hallmark of exercise‐induced skeletal muscle remodeling. For this process, considerable evidence underscores the involvement of transcriptional coactivators in mediating mitochondrial biogenesis. However, our knowledge regarding the role of transcriptional corepressors is lacking. In this study, we assessed the association of the transcriptional corepressor Rb family proteins, Rb and p107, with endurance exercise‐induced mitochondrial adaptation in human skeletal muscle. We showed that p107, but not Rb, protein levels decrease by 3 weeks of high‐intensity interval training. This is associated with significant inverse association between p107 and exercise‐induced improved mitochondrial oxidative phosphorylation. Indeed, p107 showed significant reciprocal correlations with the protein contents of representative markers of mitochondrial electron transport chain complexes. These findings in human skeletal muscle suggest that attenuated transcriptional repression through p107 may be a novel mechanism by which exercise stimulates mitochondrial biogenesis following exercise.

## Introduction

Skeletal muscle is a highly plastic tissue having the ability to undergo functional adaptations in response to external stimuli including exercise, which is known to improve metabolic health and enhance physical performance (Fluck and Hoppeler 2003; Coffey and Hawley 2007; Gundersen 2011). Exercise‐induced skeletal muscle adaptations are manifested by changes in metabolic function (Green et al. 1992) that include increases in the content and oxidative capacity of mitochondria (Holloszy 1967; Holloszy and Coyle 1984). Ultimately, the enhanced oxidative capacity of skeletal muscle is believed to contribute to more rapid activation of mitochondria oxidative phosphorylation (OXPHOS), improved fat oxidation, and carbohydrate sparing which are hallmarks of greater endurance performance (Holloszy and Coyle 1984).

Leading models propose that the greater mitochondrial oxidative capacity following chronic exercise result from continual and coordinated stimulation of transcriptional pathways. This process results in a steady state accumulation of mitochondrial proteins manifesting as greater mitochondrial content (Pilegaard et al. 2000; Fluck and Hoppeler 2003; Perry et al. 2010). Indeed, the majority of the literature has focused on the role of transcriptional coactivators and transcription factors required for promoter activation. For example, one model suggests that exercise activates peroxisome proliferator gamma coactivator‐1*α* (Pgc‐1*α*), which coactivates a variety of transcription factors controlling the expression of distinct families of mitochondrial proteins (Kupr and Handschin 2015). Surprisingly, there is very little knowledge on the role of transcriptional corepressors in mediating mitochondrial biogenesis following exercise. Conceivably, exercise might suppress transcriptional repressors themselves as part of the early stages of mitochondrial biogenesis in the course of training. Receptor interacting protein 140 (RIP140), a transcriptional corepressor of nuclear receptors regulating metabolic genes (Powelka et al. 2006) and negative regulator of skeletal muscle oxidative capacity (Seth et al. 2007) did not change in content following several weeks of exercise training in rats (Frier et al. 2011; Hoshino et al. 2013). However, in a human study with acute exercise, protein levels of RIP140 were less induced after exercise compared to a time‐matched nonexercised control group (Gidlund et al. 2015).

Recent investigations have revealed that the transcriptional corepressors, Rb (Rb1) and p107 (Rbl1) members of the retinoblastoma susceptibility (Rb) family of proteins, may represent a new mechanism by which muscle metabolic capacities are controlled through negative regulation (Fajas 2013). In addition to their typical role in cell proliferation and cell cycle, Rb was shown to promote mitochondrial integrity by inhibiting autophagy in myotubes to maintain the differentiated state (Ciavarra and Zacksenhaus 2010). Moreover, oxidative genes in skeletal muscle are repressed by Rb interaction with transcription factor E2F1 (Blanchet et al. 2011). Indeed, skeletal muscle of Rb haploinsufficient mice showed increased fatty acid uptake and oxidation compared to its wild‐type littermates (Petrov et al. 2016). In line with this, knockdown of Rb in myotubes showed enhanced mitochondrial to nuclear DNA ratio, increased oxygen consumption, increased basal glucose uptake, and disposal (Petrov et al. 2016). Also, p107 null mice have enhanced whole body lipid utilization, significantly increased levels of Pgc‐1*α* in skeletal muscle, and increased levels of oxidative muscle fiber types (Scime et al. 2005, 2010).

Despite the role of Rb and p107 in influencing skeletal muscle homeostasis, very few papers have assessed their potential influence during exercise adaptation. Rb null mice showed increased running endurance and enhanced oxidative gene expression (Blanchet et al. 2011). Moreover, after 7 days of acute exercise in mice, Rb is inactivated by phosphorylation, which might be one of the contributing factors to increase mitochondrial oxidative metabolism (Petrov et al. 2016). For humans, the role of Rb and p107 in exercise‐induced muscle adaptation has not been studied until now. Indeed, these corepressors may reveal a novel role for transcriptional de‐repression in exercise‐induced mitochondrial biogenesis distinct from the well‐established concept of transcriptional coactivation. The purpose of this study was to describe the response of Rb and p107 protein expression in human skeletal muscle following repeated intense exercise challenges. We employed a time‐course design whereby early changes in markers of skeletal muscle oxidative capacity were related to the expression of Rb and p107 transcriptional corepressors. Our findings reveal that reductions in p107 may be a novel mechanism involved in mitochondrial biogenesis following exercise in human skeletal muscle.

## Methods

### Human participants – exercise testing and muscle biopsies

All experimental procedures with human participants were approved by the Research Ethics Board at York University (Toronto, ON, Canada) and conformed to the Declaration of Helsinki. In the participants, exercise testing and muscle biopsy procedure were previously performed and described by Ydfors et al. (2016). The participants were healthy men with: mean ± SEM age of 24.8 ± 1.0 years, height 180.4 ± 1.8 cm, weight 75.5 ± 3.4 kg, body mass index of 23.2 ± 0.8 kg m^−2^ and peak oxygen uptake peak of 51.9 ± 1.9 mL kg^−1 ^min^−1^. Over 3 weeks, the subjects performed nine sessions of high‐intensity interval training (HIIT) that included 10 × 4 min intervals at 91% maximal heart rate with intermittent 2‐min rest between each interval. Before the first exercise session (T1) and preceding exercise session 5 (T5) and session 9 (T9), skeletal muscle sample was collected from vastus lateralis muscle using a percutaneous needle biopsy technique with a 14‐gauge Medax Biofeather disposable needle (San Possidonio, MO, Italy) under local anesthesia. The tissues were frozen in liquid nitrogen and stored for gene and protein expression analysis.

### OXPHOS content and individual ETC complex determination

The individual mitochondrial complex determination were previously performed, supplied and determined by Ydfors et al. (2016). The proteins of individual electron transport chain (ETC) complexes were detected by western blot analysis using a human OXPHOS Cocktail (ab110411, Abcam) containing five monoclonal antibodies specific for complex I subunit NDUFB8, complex II subunit SDHB, complex III subunit UQCRC2, complex IV subunit COX II, and complex V subunit ATP5A. The protein levels were quantified by densitometry for three time points including, before the first HIIT session (T1) and pre‐session 5 (T5) and pre‐session 9 (T9) (Ydfors et al. 2016). We calculated the total OXPHOS content for each time point by taking the sum of the protein densities for the five proteins above. The proteins were normalized with the internal control *β*‐tubulin (T8328, Sigma).

### qPCR analysis

For quantification of Rb (Rb1) and p107 (Rbl1) mRNA, reverse transcriptase polymerase chain reaction was performed using TaqMan^®^ Fast Universal PCR Master Mix (2X), no AmpErase^®^ UNG (Applied Biosystems) on the CFX384 real‐time PCR detection system (Bio‐rad). The reaction volume was 10 *μ*L, including 2 *μ*L of sample cDNA diluted 1:100, 5 *μ*L of Master Mix, 0.5 *μ*L of target‐specific probe for Rb and p107 (Hs01078066_m1 and Hs00765700_m1, Applied Biosystems), and 2.5 *μ*L nuclease‐free water. GAPDH (4352934E, Applied Biosystems) was used as an endogenous control.

### Western blot analysis

An aliquot of 10–30 mg of frozen muscle from each of the biopsies on T1, T5, and T9 were homogenized in ice‐cold buffer containing 40 mmol/L Hepes pH 7.1, 120 mmol/L NaCl, 1 mmol/L EDTA, 10 mmol/L NaHP_2_O7.10H_2_O pyrophosphate, 10 mmol/L *β*‐glycerophosphate, 10 mmol/L NaF, and 0.3% CHAPS detergent (pH 7.1). Denatured protein lysates (40 *μ*g) were loaded on gradient gels (6–15%) and separated by electrophoresis. Proteins were transferred on a 0.45 a polyvinylidene diflouride (PVDF) membrane (Bio‐rad) using a wet transfer method. The PVDF membranes were probed with rabbit polyclonal anti‐p107 (C18, SantaCruz), validated by LeCouter et al. (1998), mouse monoclonal anti‐Rb (G3‐245, BD Biosciences) validated by Huh et al. (2004), and monoclonal anti‐*α*‐tubulin (T6199, Sigma). Secondary antibodies conjugated with horseradish peroxidase were goat anti‐rabbit and anti‐mouse (Bio‐rad). The membranes were visualized with chemiluminescence on photographic films and evaluated through densitometry using Image J software.

### Correlation analysis

Correlation of p107 or Rb protein levels with the total OXPHOS content and individual mitochondrial complexes from Ydfors et al. (2016) on T1, T5, and T9 was done using Pearson correlation and linear regression analysis with Graphpad Prism 5.

### Statistical analysis

Statistical analysis was performed by Graphpad Prism 5. One‐way ANOVA and Tukey's post hoc tests were used to compare the protein contents of Rb and p107 on T1, T5, and T9. Results were considered to be statistically significant when *P *<* *0.05. Associations between variables were investigated using Pearson correlation and linear regression analysis. Statistical significance was accepted at *P *<* *0.05.

## Results

### Exercise decreases p107 protein levels in human skeletal muscle

**Figure 1:**
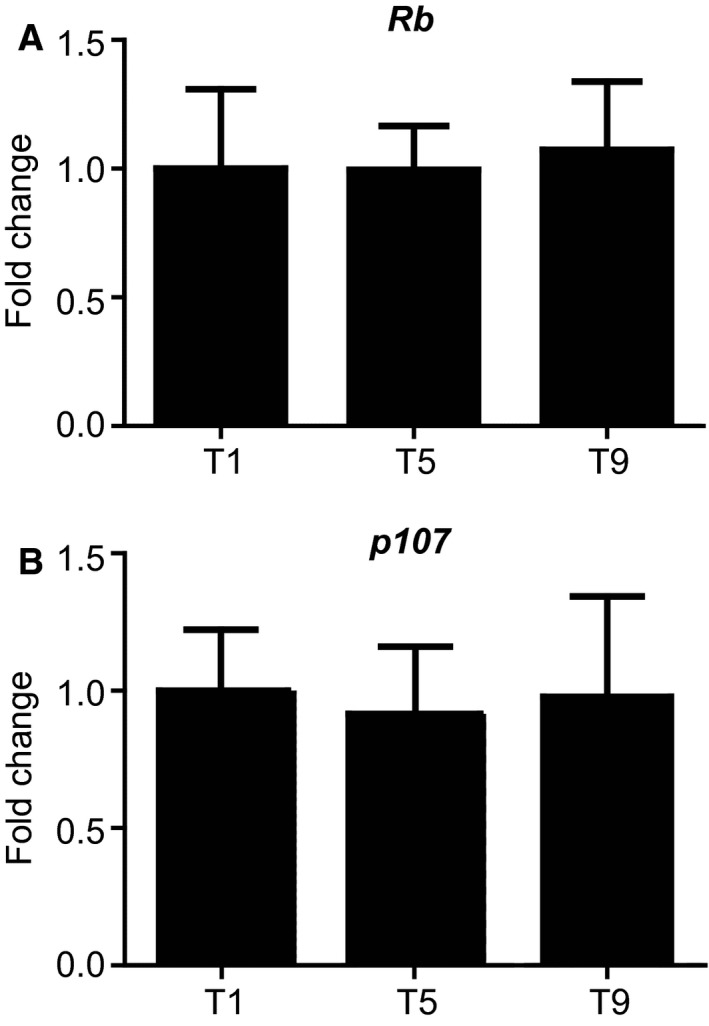
**Rb and p107 gene expressions are unaffected after nine sessions of HIIT in human skeletal muscle**. (A) qPCR analysis of Rb and (B) p107 of vastus lateralis muscle from human subjects engaged in HIIT before the initiation of the exercise protocol at T1, and pre‐exercise of session 5 (T5) and session 9 (T9). One‐way ANOVA and Tukey's post hoc test analysis were performed to determine the significance, *n* = 10 for Rb and 11 for p107. All data are presented as mean +/‐ SD.

**Figure 2:**
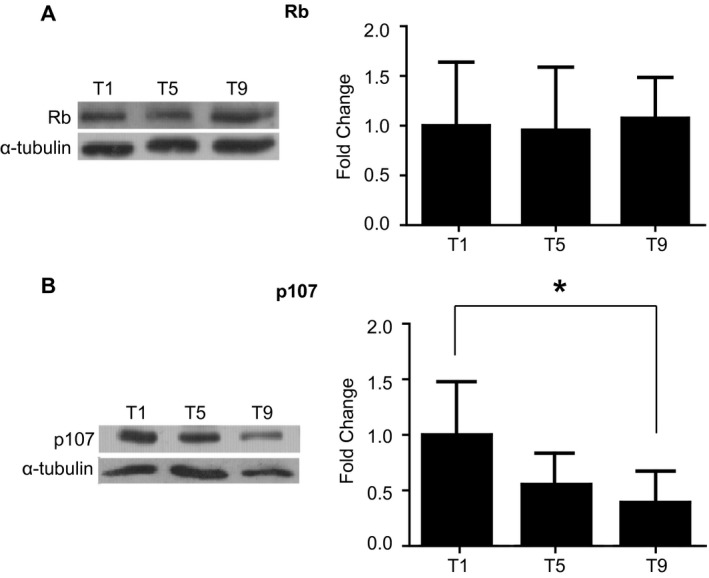
**p107 protein levels are significantly decreased over nine sessions of HIIT in human skeletal muscle**. (A) Representative western blot and graphical representation for Rb and *α*‐tubulin (control) from vastus lateralis muscle of human subjects engaged in HIIT over nine sessions before the initiation of the exercise protocol on T1 and pre‐exercise at T5 and T9. *n* = 8, one‐way ANOVA and Tukey's post hoc test analysis were performed to determine the significance. All data are +/‐ SD. (B) Representative western blot and graphical representation for p107 and *α*‐tubulin (control) as in (A). *n* = 8 (except *n* = 7 for T5), one‐way ANOVA and Tukey's post hoc test were performed to determine the significance, asterisks denote significance *P <* *0.05. All data are presented as mean +/‐ SD.

We assessed the influence of exercise‐induced skeletal muscle adaptation on Rb and p107 in human participants from a previous study reporting that markers of mitochondrial content and function increased during training (Ydfors et al. 2016). The participants were engaged in high‐intensity interval training (HIIT) over a 3‐week period, and muscle samples were obtained pre‐exercise at three consecutive time points. Gene expression analysis revealed no changes in the expression patterns of Rb and p107 in skeletal muscle biopsies collected before initiation of the exercise protocol at time point 1 (T1), and pre‐exercise at sessions 5 (T5) and 9 (T9) (Fig. 1A and B). As there were no changes for Rb and p107 in gene expression, we determined the protein levels on the biopsied samples by Western blot analysis. Unlike the change in Rb that is seen in mice in 7 days of acute exercise (Petrov et al. 2016), no change was evident in the protein expression level of Rb in the pre‐exercise samples over the entire exercise regimen in humans (Fig. 2A). However, Western blot analysis and subsequent quantification of protein levels revealed that p107 was modulated over the 3‐week period of the training session (Fig. 2B). Down‐regulation of the p107 protein level was observed in the pre‐exercise samples on T5 of exercise by 45% compared to T1 before initiating HIIT. The level of p107 decreased significantly (*P *<* *0.05) further by T9 in the pre‐exercise samples by as much as 61%. Taken together, these results suggest that 3 weeks of HIIT reduced protein expression level of p107, but not Rb in human skeletal muscle.

### p107 protein levels are inversely correlated with mitochondrial OXPHOS content

**Figure 3:**
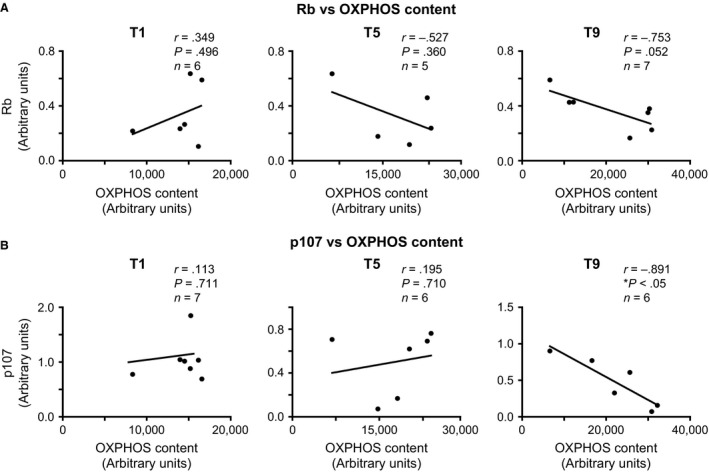
**p107 is inversely correlated with total mitochondrial OXPHOS content after nine sessions of HIIT in human skeletal muscle**. (A) Correlation and linear regression analysis between the protein levels of Rb and total OXPHOS content at T1 before the initiation of HIIT and pre‐exercise T5 and T9,. (B) Correlation and linear regression analysis between the protein levels of p107 and total mitochondria OXPHOS content as above. n's are stated on the graph, asterisks denote significance *P <* *0.05. *r* = Pearson correlation coefficient.

We investigated the potential role of Rb and p107 in muscle metabolic adaptation by assessing their relationship with the total OXPHOS content (Fig. 3A and B). This was determined by quantifying the protein expression of representative markers of the electron transport chain (ETC) at T1, T5, and T9 of HIIT (Ydfors et al. 2016). Pearson correlation and linear regression analysis revealed that p107 (*r* = 0.113, *P *=* *0.711) and Rb (*r* = 0.349, *P *=* *0.496) protein content did not correlate with OXPHOS content before the initiation of HIIT (Fig. 3A and B**)**. Interestingly, at T9 of HIIT, we found a significantly strong inverse correlation between OXPHOS content and p107 (*r* = −0.891, *P *<* *0.05) (Fig. 3B). On the contrary, Rb failed to show any significant correlation with the total OXPHOS content at T9 (Fig. 3A). These data suggest that the improved mitochondrial oxidative capacity associated with exercise have a strong inverse association with the transcriptional corepressor p107 but not Rb.

### p107 and Rb protein levels are inversely correlated with mitochondrial ETC complexes

**Figure 4:**
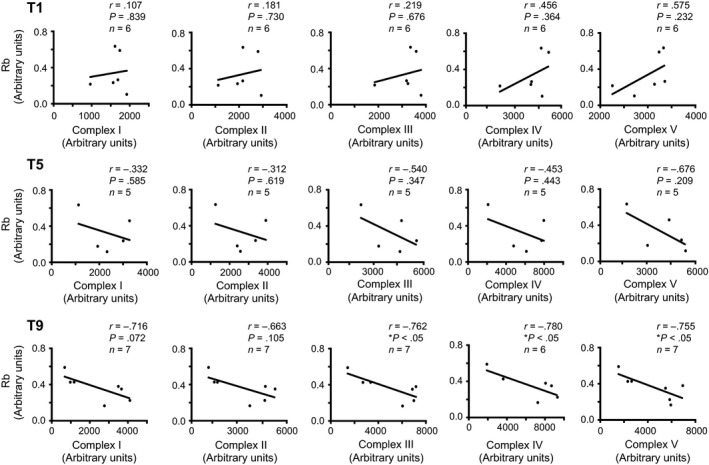
**Rb is inversely correlated with ETC complex III, IV and V after nine sessions of HIIT in human skeletal muscle**. Correlation and linear regression analysis between the protein levels of Rb and complex I, complex II, complex III, complex IV, or complex V of the ETC at T1, T5, and T9 of HIIT. n's are stated on the graph, asterisks denote significance *P <* *0.05. *r* = Pearson correlation coefficient. HIIT, high‐intensity interval training.

**Figure 5:**
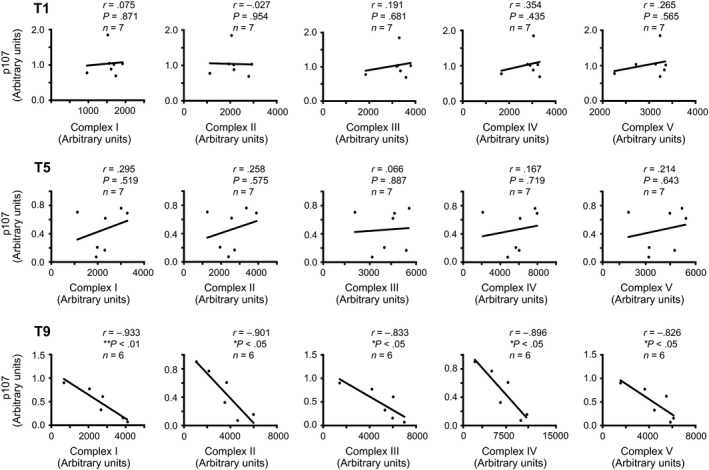
**p107 is inversely correlated with individual ETC complexes after nine sessions of HIIT in human skeletal muscle**. Correlation and linear regression analysis between the protein levels of p107 and complex I, complex II, complex III, complex IV, or complex V of the ETC at T1, T5, and T9 of HIIT. n's are stated on the graph, asterisks denote significance *P <* *0.05 and **P <* *0.01. *r* = Pearson correlation coefficient. HIIT, high‐intensity interval training.

As a significant association was found between the total OXPHOS content and p107, we evaluated if the mitochondrial ETC complexes separately had correlation with Rb and p107. This was accomplished by comparing the protein content of a representative marker for each of the five mitochondrial ETC complexes with Rb or p107. Before the onset of the HIIT regimen at T1 as well as at T5, we found that neither p107 nor Rb showed correlation with any of the ETC complexes (Figs. 4, 5). In line with the correlation between the total OXPHOS content and p107 at T9, a very strong negative correlation was also present between every ETC complex and p107 (Fig. 5). Although Rb showed no significant association with the total OXPHOS content on T9, a significant negative correlation was observed only with Complexes III, IV, and V (Fig. 4). This suggests that p107, and to a minor extent Rb, establishes an intricate association with the enhanced mitochondrial content caused by exercise.

## Discussion

The adaptation of skeletal muscle in response to exercise has been a matter of extensive research, as considerable evidence exists for the beneficial effects of exercise for improving adverse health conditions (Schenk and Horowitz 2007). Our study used nine sessions of HIIT over a 3‐week period on human subjects as a model for acute endurance exercise to assess its influence on the transcriptional corepressors, Rb and p107. HIIT is a powerful model for investigating muscle plasticity given that it challenges metabolic homeostasis through repetitive intense bursts of contraction.

At present it is not known if p107 targets any of the nuclear or mitochondrial genes promoters associated with OXPHOS. However, the concept of the Rb family interacting with metabolic gene promoters is not atypical as Jones et al. (2016) found that Rb loss in triple‐negative breast cancer induces mitochondrial protein translation, concomitant with increased OXPHOS. Although, others have found that Rb loss has the opposite effect by repressing OXPHOS in noncancerous cells (Nicolay et al. 2015). In addition, an early report, using ChIP‐seq, demonstrated the association of E2F4 (a transcription factor binding partner of the RB family) on gene promoters involved with mitochondrial biogenesis (Cam et al. 2004). However, out of more than 10,000 E2F4 binding sites that have been identified through ChIP‐seq analysis of E2F4, none has been specifically annotated for mitochondrial biogenesis (Cam et al. 2004; Balciunaite et al. 2005; Lee et al. 2011). Additionally, a large number of E2F4 targets specifically interact without p107 (Balciunaite et al. 2005) and p107 is known to interact with several other transcription factors including Sp1, b‐myb, c‐myc, and Smad3 (Balciunaite et al. 2005; Wirt and Sage 2010). A p107‐specific ChIP analysis of a DNA microarray (ChIP on ChIP), containing 13,000 human gene promoter regions spanning 700 bp upstream and 200 bp downstream of the transcription start sites, revealed that p107 was bound to 244 genes, among which some are involved in mitochondrial biogenesis (Balciunaite et al. 2005). To date, none of these promoters involved in mitochondrial biogenesis have been annotated for specific p107 binding using p107 genetically deleted cells as controls. Nonetheless, the increased expression patterns for these genes based on potential p107 de‐repression during HIIT might reflect the involvement of another factor(s) that is controlled by p107.

Our data suggest a novel association of p107 with the mitochondrial adaptations that are established after nine sessions of HIIT over 3 weeks. Indeed, the novel findings of this study are that, by the end of the exercise regimen there was (1) a decrease in p107 protein levels, (2) a strong inverse association of p107 with OXPHOS content, and (3) an inverse relation of p107 with each of the five ETC complexes. Therefore, these findings provide a newly discovered association between transcriptional corepressor down‐regulation with increased mitochondrial bioenergetics.

Many findings show that p107 levels are normally controlled transcriptionally. Few details are known regarding posttranslational regulation of p107 levels. Two early reports suggest that Ca^2+^‐activated protease calpain and the ubiquitin‐proteasome pathway might posttranslationally regulate p107 levels (Jang et al. 1999). Intriguingly, during running exercise, total calpain activity is increased and is associated with enhanced rates of protein degradation that is known to occur in skeletal muscle soon after exercise (Belcastro et al. 1998). Future studies investigating if the increase in calpain activity during exercise adaptation would target p107 for degradation are required.

Our findings strongly suggest that Rb might not have a role in influencing skeletal muscle adaptation triggered by exercise at the end of HIIT. Intriguingly, we found there were no changes in the transcriptional and posttranscriptional levels of Rb. Also there were no apparent differences in posttranslational modification by phosphorylation, as determined by the mobility of Rb through gels, which was unaltered on Western blots. However, p107 showed a significant inverse correlation with each of the five ETC complexes and total OXPHOS content when sum of the complexes were assessed. This is in contrast to Rb that showed negative association with only complexes III, IV, and V and did not show any significant relation when compared to total OXPHOS. In mice, 7 days of acute exercise inactivates Rb in skeletal muscle by its phosphorylation by cyclin‐dependent kinases (Petrov et al. 2016). The inactivation of Rb is synonymous with enhanced fatty acid oxidation, glucose uptake, and mitochondrial fusion (Petrov et al. 2016). These metabolic manifestations are the same as observed in human skeletal muscle after exercise training (Holloszy and Coyle 1984; Neufer and Dohm 1993; Cartoni et al. 2005; Talanian et al. 2007; Perry et al. 2008; Richter and Hargreaves 2013). Thus, it remains to be determined if Rb inactivation by phosphorylation or other posttranslational modification(s) is associated with these metabolic improvements following training. In addition, the differences in the mouse and our human study for Rb might be species‐specific and/or a disparity with the exercise methodologies used.

In mouse, it was shown that knockdown and knockout of p107 myoblasts resulted in more oxidative fiber types (Scime et al. 2010). Interestingly, 6 weeks of HIIT also has been shown to induce myoblast activation followed by its differentiation into more metabolically efficient fiber types in humans (Joanisse et al. 2013). However, our human subjects did not show any fiber‐type remodeling after nine sessions of HIIT despite a decrease in the p107 levels and an increase in skeletal muscle oxidative capacities, as published previously in these participants (Ydfors et al. 2016). This discrepancy might be explained by the differences in the duration of the HIIT regimen between the two studies, which might play a crucial role in dictating the fate of myoblasts in humans.

There is strong evidence that inverse association between p107 and the mitochondrial metabolic machinery might be due to a functional role for p107. It has been shown that down‐regulation of p107 is associated with oxidative phenotypes such as slow fiber formation (Scime et al. 2010) and brown fat formation (Scime et al. 2005; De Sousa et al. 2014) in mice. Indeed, in mouse skeletal muscle, p107 has been shown to suppress the transcriptional levels of Pgc‐1*α*, a master regulator of exercise‐induced mitochondrial biogenesis, through direct promoter interaction (Scime et al. 2010). In humans, several weeks of endurance training (Russell et al. 2003; Short et al. 2003; Kuhl et al. 2006) and acute exercise (Pilegaard et al. 2003; Norrbom et al. 2004; Watt et al. 2004; Cartoni et al. 2005; Russell et al. 2005; Hellsten et al. 2007; Mortensen et al. 2007) are sufficient to increase Pgc‐1*α* mRNA. This suggests that the decrease in p107 levels might release repression on Pgc‐1*α* promoter. Perry et al. (2010) showed that following every session of HIIT, Pgc‐1*α* mRNA increased 4 h post exercise. However, Pgc‐1*α* returned to its pre‐exercise levels after 24 h. We did not test samples for p107 immediately after exercise to assess if its levels were reduced to account for the increased Pgc‐1*α* mRNA immediately post exercise.

In summary, we have found an association between a transcriptional corepressor with improved oxidative capacity after exercise adaptation. Indeed, in human skeletal muscle, p107 protein content is decreased concurrent with increased markers of mitochondrial content during short‐term HIIT. This negative relationship suggests that the classic models of regulating mitochondrial biogenesis through positive coactivation should consider the potential role of decreasing transcriptional repression. Moreover, our results highlight that p107 might be more important than Rb in human skeletal muscle adaptation during exercise. Given the clear relationship between skeletal muscle oxidative capacity and metabolic health, elucidating the role of transcriptional de‐repression is of great importance in unraveling the mechanisms by which exercise mediates protection from metabolic disease.

## Conflict of Interest

None declared.

## References

[phy213155-bib-0001] Balciunaite, E. , A. Spektor , N. H. Lents , H. Cam , H. Te Riele , A. Scime , et al. 2005 Pocket protein complexes are recruited to distinct targets in quiescent and proliferating cells. Mol. Cell. Biol. 25:8166–8178. 16135806 10.1128/MCB.25.18.8166-8178.2005 PMC1234327

[phy213155-bib-0002] Belcastro, A. N. , L. D. Shewchuk , and D. A. Raj . 1998 Exercise‐induced muscle injury: a calpain hypothesis. Mol. Cell. Biochem. 179:135–145. 9543356 10.1023/a:1006816123601

[phy213155-bib-0003] Blanchet, E. , J. S. Annicotte , S. Lagarrigue , V. Aguilar , C. Clape , C. Chavey , et al. 2011 E2F transcription factor‐1 regulates oxidative metabolism. Nat. Cell Biol. 13:1146–1152. 21841792 10.1038/ncb2309 PMC3849758

[phy213155-bib-0004] Cam, H. , E. Balciunaite , A. Blais , A. Spektor , R. C. Scarpulla , R. Young , et al. 2004 A common set of gene regulatory networks links metabolism and growth inhibition. Mol. Cell 16:399–411. 15525513 10.1016/j.molcel.2004.09.037

[phy213155-bib-0005] Cartoni, R. , B. Leger , M. B. Hock , M. Praz , A. Crettenand , S. Pich , et al. 2005 Mitofusins 1/2 and ERRalpha expression are increased in human skeletal muscle after physical exercise. J. Physiol. 567:349–358. 15961417 10.1113/jphysiol.2005.092031 PMC1474174

[phy213155-bib-0006] Ciavarra, G. , and E. Zacksenhaus . 2010 Rescue of myogenic defects in Rb‐deficient cells by inhibition of autophagy or by hypoxia‐induced glycolytic shift. J. Cell Biol. 191:291–301. 20937698 10.1083/jcb.201005067 PMC2958467

[phy213155-bib-0007] Coffey, V. G. , and J. A. Hawley . 2007 The molecular bases of training adaptation. Sports Med. 37:737–763. 17722947 10.2165/00007256-200737090-00001

[phy213155-bib-0008] De Sousa, M. , D. P. Porras , C. G. Perry , P. Seale , and A. Scime . 2014 p107 is a crucial regulator for determining the adipocyte lineage fate choices of stem cells. Stem Cells 32:1323–1336. 24449206 10.1002/stem.1637

[phy213155-bib-0009] Fajas, L. 2013 Re‐thinking cell cycle regulators: the cross‐talk with metabolism. Front Oncol. 3:4. 23355973 10.3389/fonc.2013.00004 PMC3555080

[phy213155-bib-0010] Fluck, M. , and H. Hoppeler . 2003 Molecular basis of skeletal muscle plasticity–from gene to form and function. Rev. Physiol. Biochem. Pharmacol. 146:159–216. 12605307 10.1007/s10254-002-0004-7

[phy213155-bib-0011] Frier, B. C. , C. R. Hancock , J. P. Little , N. Fillmore , T. A. Bliss , D. M. Thomson , et al. 2011 Reductions in RIP140 are not required for exercise‐ and AICAR‐mediated increases in skeletal muscle mitochondrial content. J. Appl. Physiol. (1985) 111:688–695. 21700896 10.1152/japplphysiol.00279.2011

[phy213155-bib-0012] Gidlund, E. K. , M. Ydfors , S. Appel , H. Rundqvist , C. J. Sundberg , and J. Norrbom . (2015). Rapidly elevated levels of PGC‐1alpha‐b protein in human skeletal muscle after exercise: exploring regulatory factors in a randomized controlled trial. J. Appl. Physiol. (1985) 119: 374–384. 26089547 10.1152/japplphysiol.01000.2014

[phy213155-bib-0013] Green, H. J. , R. Helyar , M. Ball‐Burnett , N. Kowalchuk , S. Symon , and B. Farrance . (1992). Metabolic adaptations to training precede changes in muscle mitochondrial capacity. J. Appl. Physiol. (1985) 72: 484–491. 1559923 10.1152/jappl.1992.72.2.484

[phy213155-bib-0014] Gundersen, K. 2011 Excitation‐transcription coupling in skeletal muscle: the molecular pathways of exercise. Biol. Rev. Camb. Philos. Soc. 86:564–600. 21040371 10.1111/j.1469-185X.2010.00161.x PMC3170710

[phy213155-bib-0015] Hellsten, Y. , J. J. Nielsen , J. Lykkesfeldt , M. Bruhn , L. Silveira , H. Pilegaard , et al. 2007 Antioxidant supplementation enhances the exercise‐induced increase in mitochondrial uncoupling protein 3 and endothelial nitric oxide synthase mRNA content in human skeletal muscle. Free Radic. Biol. Med. 43:353–361. 17602951 10.1016/j.freeradbiomed.2007.02.029

[phy213155-bib-0016] Holloszy, J. O. 1967 Biochemical adaptations in muscle. Effects of exercise on mitochondrial oxygen uptake and respiratory enzyme activity in skeletal muscle. J. Biol. Chem. 242:2278–2282. 4290225

[phy213155-bib-0017] Holloszy, J. O. , and E. F. Coyle . 1984 Adaptations of skeletal muscle to endurance exercise and their metabolic consequences. J. Appl. Physiol. Respir. Environ. Exerc. Physiol. 56:831–838. 6373687 10.1152/jappl.1984.56.4.831

[phy213155-bib-0018] Hoshino, D. , Y. Yoshida , Y. Kitaoka , H. Hatta , and A. Bonen . 2013 High‐intensity interval training increases intrinsic rates of mitochondrial fatty acid oxidation in rat red and white skeletal muscle. Appl. Physiol. Nutr. Metab. 38:326–333. 23537026 10.1139/apnm-2012-0257

[phy213155-bib-0019] Huh, M. S. , M. H. Parker , A. Scime , R. Parks , and M. A. Rudnicki . 2004 Rb is required for progression through myogenic differentiation but not maintenance of terminal differentiation. J. Cell Biol. 166:865–876. 15364961 10.1083/jcb.200403004 PMC2172111

[phy213155-bib-0020] Jang, J. S. , S. J. Lee , Y. H. Choi , P. M. Nguyen , J. Lee , S. G. Hwang , et al. 1999 Posttranslational regulation of the retinoblastoma gene family member p107 by calpain protease. Oncogene 18:1789–1796. 10086333 10.1038/sj.onc.1202497

[phy213155-bib-0021] Joanisse, S. , J. B. Gillen , L. M. Bellamy , B. R. McKay , M. A. Tarnopolsky , M. J. Gibala , et al. 2013 Evidence for the contribution of muscle stem cells to nonhypertrophic skeletal muscle remodeling in humans. FASEB J. 27:4596–4605. 23928822 10.1096/fj.13-229799 PMC3804745

[phy213155-bib-0022] Jones, R. A. , T. J. Robinson , J. C. Liu , M. Shrestha , V. Voisin , Y. Ju , et al. 2016 RB1 deficiency in triple‐negative breast cancer induces mitochondrial protein translation. J. Clin. Invest. 126:3739–3757. 27571409 10.1172/JCI81568 PMC5096803

[phy213155-bib-0023] Kuhl, J. E. , N. B. Ruderman , N. Musi , L. J. Goodyear , M. E. Patti , S. Crunkhorn , et al. 2006 Exercise training decreases the concentration of malonyl‐CoA and increases the expression and activity of malonyl‐CoA decarboxylase in human muscle. Am. J. Physiol. Endocrinol. Metab. 290:E1296–E1303. 16434556 10.1152/ajpendo.00341.2005

[phy213155-bib-0024] Kupr, B. , and C. Handschin . 2015 Complex coordination of cell plasticity by a pgc‐1alpha‐controlled transcriptional network in skeletal muscle. Front. Physiol. 6:325. 26617528 10.3389/fphys.2015.00325 PMC4639707

[phy213155-bib-0025] LeCouter, J. E. , B. Kablar , W. R. Hardy , C. Ying , L. A. Megeney , L. L. May , et al. 1998 Strain‐dependent myeloid hyperplasia, growth deficiency, and accelerated cell cycle in mice lacking the Rb‐related p107 gene. Mol. Cell. Biol. 18:7455–7465. 9819431 10.1128/mcb.18.12.7455 PMC109326

[phy213155-bib-0026] Lee, B. K. , A. A. Bhinge , and V. R. Iyer . 2011 Wide‐ranging functions of E2F4 in transcriptional activation and repression revealed by genome‐wide analysis. Nucleic Acids Res. 39:3558–3573. 21247883 10.1093/nar/gkq1313 PMC3089461

[phy213155-bib-0027] Mortensen, O. H. , P. Plomgaard , C. P. Fischer , A. K. Hansen , H. Pilegaard , and B. K. Pedersen . 2007 PGC‐1beta is downregulated by training in human skeletal muscle: no effect of training twice every second day vs. once daily on expression of the PGC‐1 family. J. Appl. Physiol. (1985) 103: 1536–1542. 17690194 10.1152/japplphysiol.00575.2007

[phy213155-bib-0028] Neufer, P. D. , and G. L. Dohm . 1993 Exercise induces a transient increase in transcription of the GLUT‐4 gene in skeletal muscle. Am. J. Physiol. 265:C1597–C1603. 7506491 10.1152/ajpcell.1993.265.6.C1597

[phy213155-bib-0029] Nicolay, B. N. , P. S. Danielian , F. Kottakis , J. D. Jr Lapek , I. Sanidas , W. O. Miles , et al. 2015 Proteomic analysis of pRb loss highlights a signature of decreased mitochondrial oxidative phosphorylation. Genes Dev. 29:1875–1889. 26314710 10.1101/gad.264127.115 PMC4573859

[phy213155-bib-0030] Norrbom, J. , C. J. Sundberg , H. Ameln , W. E. Kraus , E. Jansson , and T. Gustafsson . 2004 PGC‐1alpha mRNA expression is influenced by metabolic perturbation in exercising human skeletal muscle. J. Appl. Physiol. (1985) 96: 189–194. 12972445 10.1152/japplphysiol.00765.2003

[phy213155-bib-0031] Perry, C. G. , G. J. Heigenhauser , A. Bonen , and L. L. Spriet . 2008 High‐intensity aerobic interval training increases fat and carbohydrate metabolic capacities in human skeletal muscle. Appl. Physiol. Nutr. Metab. 33:1112–1123. 19088769 10.1139/H08-097

[phy213155-bib-0032] Perry, C. G. , J. Lally , G. P. Holloway , G. J. Heigenhauser , A. Bonen , and L. L. Spriet . 2010 Repeated transient mRNA bursts precede increases in transcriptional and mitochondrial proteins during training in human skeletal muscle. J. Physiol. 588:4795–4810. 20921196 10.1113/jphysiol.2010.199448 PMC3010147

[phy213155-bib-0033] Petrov, P. D. , J. Ribot , I. C. Lopez‐Mejia , L. Fajas , A. Palou , and M. L. Bonet . 2016 Retinoblastoma Protein Knockdown Favors Oxidative Metabolism and Glucose and Fatty Acid Disposal in Muscle Cells. J. Cell. Physiol. 231:708–718. 26241807 10.1002/jcp.25121

[phy213155-bib-0034] Pilegaard, H. , G. A. Ordway , B. Saltin , and P. D. Neufer . 2000 Transcriptional regulation of gene expression in human skeletal muscle during recovery from exercise. Am. J. Physiol. Endocrinol. Metab. 279:E806–E814. 11001762 10.1152/ajpendo.2000.279.4.E806

[phy213155-bib-0035] Pilegaard, H. , B. Saltin , and P. D. Neufer . 2003 Exercise induces transient transcriptional activation of the PGC‐1alpha gene in human skeletal muscle. J. Physiol. 546:851–858. 12563009 10.1113/jphysiol.2002.034850 PMC2342594

[phy213155-bib-0036] Powelka, A. M. , A. Seth , J. V. Virbasius , E. Kiskinis , S. M. Nicoloro , A. Guilherme , et al. 2006 Suppression of oxidative metabolism and mitochondrial biogenesis by the transcriptional corepressor RIP140 in mouse adipocytes. J. Clin. Invest. 116:125–136. 16374519 10.1172/JCI26040 PMC1319222

[phy213155-bib-0037] Richter, E. A. , and M. Hargreaves . 2013 Exercise, GLUT4, and skeletal muscle glucose uptake. Physiol. Rev. 93:993–1017. 23899560 10.1152/physrev.00038.2012

[phy213155-bib-0038] Russell, A. P. , J. Feilchenfeldt , S. Schreiber , M. Praz , A. Crettenand , C. Gobelet , et al. 2003 Endurance training in humans leads to fiber type‐specific increases in levels of peroxisome proliferator‐activated receptor‐gamma coactivator‐1 and peroxisome proliferator‐activated receptor‐alpha in skeletal muscle. Diabetes 52:2874–2881. 14633846 10.2337/diabetes.52.12.2874

[phy213155-bib-0039] Russell, A. P. , M. K. Hesselink , S. K. Lo , and P. Schrauwen . 2005 Regulation of metabolic transcriptional co‐activators and transcription factors with acute exercise. FASEB J. 19:986–988. 15814608 10.1096/fj.04-3168fje

[phy213155-bib-0040] Schenk, S. , and J. F. Horowitz . 2007 Acute exercise increases triglyceride synthesis in skeletal muscle and prevents fatty acid‐induced insulin resistance. J. Clin. Invest. 117:1690–1698. 17510709 10.1172/JCI30566 PMC1866251

[phy213155-bib-0041] Scime, A. , G. Grenier , M. S. Huh , M. A. Gillespie , L. Bevilacqua , M. E. Harper , et al. 2005 Rb and p107 regulate preadipocyte differentiation into white versus brown fat through repression of PGC‐1alpha. Cell Metab. 2:283–295. 16271529 10.1016/j.cmet.2005.10.002

[phy213155-bib-0042] Scime, A. , V. D. Soleimani , C. F. Bentzinger , M. A. Gillespie , F. Le Grand , G. Grenier , et al. 2010 Oxidative status of muscle is determined by p107 regulation of PGC‐1a. J. Cell Biol. 190:651–662. 20713602 10.1083/jcb.201005076 PMC2928004

[phy213155-bib-0043] Seth, A. , J. H. Steel , D. Nichol , V. Pocock , M. K. Kumaran , A. Fritah , et al. 2007 The transcriptional corepressor RIP140 regulates oxidative metabolism in skeletal muscle. Cell Metab. 6:236–245. 17767910 10.1016/j.cmet.2007.08.004 PMC2680991

[phy213155-bib-0044] Short, K. R. , J. L. Vittone , M. L. Bigelow , D. N. Proctor , R. A. Rizza , J. M. Coenen‐Schimke , et al. 2003 Impact of aerobic exercise training on age‐related changes in insulin sensitivity and muscle oxidative capacity. Diabetes 52:1888–1896. 12882902 10.2337/diabetes.52.8.1888

[phy213155-bib-0045] Talanian, J. L. , S. D. Galloway , G. J. Heigenhauser , A. Bonen , and L. L. Spriet . 2007 Two weeks of high‐intensity aerobic interval training increases the capacity for fat oxidation during exercise in women. J. Appl. Physiol. (1985) 102: 1439–1447. 17170203 10.1152/japplphysiol.01098.2006

[phy213155-bib-0046] Watt, M. J. , R. J. Southgate , A. G. Holmes , and M. A. Febbraio . 2004 Suppression of plasma free fatty acids upregulates peroxisome proliferator‐activated receptor (PPAR) alpha and delta and PPAR coactivator 1alpha in human skeletal muscle, but not lipid regulatory genes. J. Mol. Endocrinol. 33:533–544. 15525607 10.1677/jme.1.01499

[phy213155-bib-0047] Wirt, S. E. , and J. Sage . 2010 p107 in the public eye: an Rb understudy and more. Cell Div. 5:9. 20359370 10.1186/1747-1028-5-9 PMC2861648

[phy213155-bib-0048] Ydfors, M. , M. C. Hughes , R. Laham , U. Schlattner , J. Norrbom , and C. G. Perry . 2016 Modelling in vivo creatine/phosphocreatine in vitro reveals divergent adaptations in human muscle mitochondrial respiratory control by ADP after acute and chronic exercise. J. Physiol. 594:3127–3140. 26631938 10.1113/JP271259 PMC4887669

